# Development of a Quantitative One-Step RT-PCR Method for the Detection of Sabin 2 Virus Contamination in a Novel Oral Poliovirus Vaccine Type 2

**DOI:** 10.3390/vaccines9070688

**Published:** 2021-06-23

**Authors:** Hasmik Manukyan, Erman Tritama, Rahnuma Wahid, Azeem Ansari, John Konz, Konstantin Chumakov, Majid Laassri

**Affiliations:** 1 Division of Viral Products, Center for Biologics Evaluation and Research, US Food and Drug Administration, Silver Spring, MD 20993, USA; hasmik.manukyan@fda.hhs.gov (H.M.); konstantin.chumakov@fda.hhs.gov (K.C.); 2 Research and Development Division, PT. Bio Farma, Bandung 40161, Indonesia; erman.tritama@biofarma.co.id; 3 Center for Vaccine Innovation and Access, PATH, Seattle, WA 98121, USA; rwahid@path.org (R.W.); aansari@path.org (A.A.); jkonz@path.org (J.K.)

**Keywords:** release test, quality control, OPV, nOPV, Sabin 2 virus contaminant

## Abstract

To control circulating vaccine-derived type 2 poliovirus outbreaks, a more genetically stable novel Oral Poliovirus Vaccine type 2 (nOPV2) was developed by targeted modifications of Sabin 2 genome. Since the use of OPV2 made of Sabin 2 strain has been stopped, it is important to exclude the possibility that batches of nOPV2 are contaminated with Sabin 2 virus. Here, we report the development of a simple quantitative one-step reverse-transcription polymerase chain reaction assay for the detection and quantitation of Sabin 2 virus in the presence of overwhelming amounts of nOPV2 strain. The method is specific and linear within 8 log_10_ range even in the presence of relevant amounts of nOPV2 virus. It is sensitive, with a lower limit of detection of 0.2 CCID_50_/mL (an equivalent of 198 genome copies per mL), and generates reproducible results. This assay can be used for quality control and lot release of the nOPV2.

## 1. Introduction

There are two prophylactic vaccines against poliomyelitis caused by the three serotypes of poliovirus: inactivated poliovirus vaccine (IPV) and live oral poliovirus vaccine (OPV). The use of these vaccines has dramatically reduced polio incidence worldwide. However, IPV does not stimulate adequate mucosal immunity to prevent transmission of circulating polioviruses [1,2], and OPV is composed of three Sabin strains that are genetically unstable and can revert to neurovirulence [3,4], leading to about one case of vaccine-associated paralytic poliomyelitis (VAPP) per one million vaccine recipients.

The Global Polio Eradication Initiative (GPEI) led to the eradication of wild polioviruses of types 2 and 3 [5], but wild type-1 poliovirus remains endemic in Afghanistan and Pakistan [6]. This progress was achieved mostly because of the massive use of OPV that stimulates strong systemic and mucosal immunity [7]. The recipients of OPV excrete revertant vaccine viruses that can be serially transmitted and convert to vaccine-derived polioviruses (VDPVs) causing outbreaks in poorly immunized communities [8]. People with primary B-cell immunodeficiency can become chronically infected and excrete VDPV for a long time [9,10,11]. To prevent generation of VDPV and avoid VAPP, many high- and middle-income countries started to use IPV instead of OPV [12]. In addition, in April–May of 2016 the use of trivalent OPV was stopped globally and replaced with bivalent OPV (bOPV) that contains only serotypes 1 and 3, supplemented with at least one dose of IPV [13,14]. As stated above, IPV elicits poor mucosal immunity and thus the vaccinees receiving bOPV with IPV are at risk of being infected with and transmitting type 2 poliovirus [15,16,17]. To overcome the limitations of current vaccines, two genetically-stabilized novel OPV type 2 (nOPV2) vaccine candidates were developed [18,19]. Both candidates include an alternative domain V from the S15 strain [19] which incorporated 18 nucleotide substitutions (compared to Sabin 2 strain) aimed at avoiding thermodynamic stabilization of the structure that leads to the increase of neurovirulence. While vaccine manufacturers maintain both nOPV2 and Sabin 2 strains in their inventory, it is important to exclude the possibility of accidental Sabin 2 contamination of nOPV2 lots through a combination of manufacturing controls and quality control testing. Any test for Sabin 2 contamination in nOPV2 should be sensitive, specific and be able to discriminate Sabin 2 virus from nOPV2.

Several molecular-based procedures have been developed for detection of OPV strains, including ELISA [20], reverse transcription-polymerase chain reaction (RT-PCR) followed by hybridization with specific oligonucleotides [21,22], and quantitative RT-PCR [23] that uses degenerate primers with mixed-base and inosine residues. Recently we have developed quantitative one-step RT-PCR (qosRT-PCR) and quantitative multiplex one-step RT-PCR (qmosRT-PCR) assays for detection and quantitation of the three Sabin strains of OPV [24,25]. None of these methods were designed to specifically identify Sabin strains in the presence of nOPV. In this study we propose a simple qosRT-PCR assay using specific primers and a TaqMan probe designed to discriminate between the S15 domain V present in the nOPV2 candidates and Sabin 2 virus, thereby allowing specific detection of Sabin 2 virus in nOPV2 lots.

The assay for the presence of Sabin 2 contamination in nOPV2 stocks is simple, rapid, reproducible, sensitive, and has large linearity range suitable for quality control of the nOPV2 vaccine.

## 2. Materials and Methods

### 2.1. Viruses

Batch of US neurovirulence poliovirus reference vaccine (Sabin 2 strain of OPV having GenBank accession number AY184220) and plasmid-containing Sabin 2 genome were used as positive control (Lab. method development inventory at US. FDA). Plasmid-containing nOPV2-c1 genome with mutation E_295_K was provided by Dr. Andrew Macadam (NIBSC, UK) and Dr. Raul Andino (UCSF, US), and was used as negative control for the Sabin 2 contamination assay. Monovalent bulks of nOPV2 candidate 1 (nOPV2-c1) batches nPOL 2016 and nPOL 2018C, monovalent nOPV2 candidate 2 (nOPV2-c2) batches nPOL 2038C and nPOL 2056C, and drug product nOPV2-c1 batches 2060119C, 2060219C and 2060319C were provided by P.T. Bio Farma (Indonesia) and were tested for the presence of Sabin 2 virus.

nOPV2c1 virus was recovered from the plasmid containing nOPV2-c1 genome with mutation E_295_K by transfecting HEp-2C monolayers with T7 transcripts [19,26] and incubating them at 34 °C and 5% CO_2_ until the complete cytopathic effect was apparent. The recovered nOPV2-c1-virus was sequenced as described below and named nOPV2c1-295. It was used as negative control for the assay and in the spiking (spiking Sabin 2 virus in nOPV2c1-295) experiment (see below).

Sabin 2 and the recovered nOPV2c1-295 viruses were titrated by MPBT assay [27] with small modifications; only primers and probe for poliovirus type 2 were used.

### 2.2. Extraction of Viral RNA

Viral RNA was extracted from viral samples according to the manufacturer’s protocol of the QIAamp viral RNA mini kit (QIAGEN, Chatsworth, CA, USA). The extracted RNA was eluted in a final volume of 60 μL of sterile RNase-free water and was frozen at −80 °C for further use.

### 2.3. Quantitative One-Step RT-PCR

The quantitative one-step RT-PCR (qosRT-PCR) reactions were prepared in 96-well optical plates in a final volume of 25 μL using 2 μL of viral RNA and QuantiFast Multiplex RT-PCR Kit (QIAGEN, Valencia, CA, USA). The RNA from Sabin 2 virus with known titer (expressed as CCID_50_/mL) was subject to serial 10-fold dilution and used as reference standard to generate the standard curve (at least the concentrations of 10^6^ to 10 CCID_50_/mL of the diluted samples were run in triplicates). The Sabin 2 RNA and plasmid-containing Sabin 2 genome were used as positive control, and water, nOPV2 RNA and plasmid-containing nOPV2 genome were used as negative controls. All control samples were run in quadruplicates. The specific primers and probe used for detection and quantitation of Sabin 2 strain are presented in Table 1. Oligonucleotide probe Sab2PrbFAM2 was used at a final concentration of 25 nM, and primers Sab2-605R (reverse) and Sab2-538F (forward) were used at a final concentration of 0.8 μM. The qosRT-PCR procedure was performed using real-time PCR System ViiA7 (Thermo Fisher Scientific, South San Francisco, CA, USA) at the following thermal cycling conditions: one cycle incubation for 20 min at 50 °C and 5 min at 95 °C, followed by 40–45 cycles, each consisting of 15 s at 94 °C, 15 s at 55 °C, and 30 s at 60 °C.

### 2.4. Specificity, Limit-of-Detection and Linearity of qosRT-PCR to Detect Sabin 2 Virus

To evaluate the limit-of-detection of the qosRT-PCR, 8.34 log_10_ CCID_50_/mL of Sabin 2 virus was subjected to RNA extraction as described above, then viral RNA was serially diluted in water (10^−1^ to 10^−10^) in a total volume of 0.1 mL. Two microliters of each dilution of RNA was analyzed in triplicate by qosRT-PCR as described above.

In addition, to evaluate the ability of qosRT-PCR to specifically detect and quantify Sabin 2 virus, 2 μL of each of Sabin 2 RNA and plasmid containing Sabin 2 genome was tested as positive controls in four repeats, and as negative controls 2 μL of each of plasmid containing nOPV2 genome and water were tested in four repeats in the same plate by qosRT-PCR assay.

In a similar experiment and in the same context, Sabin2 virus (8.34 Log_10_ CCID_50_/mL) was serially spiked in nOPV2c1-295 virus (8.07 Log_10_ CCID_50_/mL) suspension as follows: twelve 1.5 mL–tubes were filled with 180 μL of nOPV2c1-295, 20 μL of Sabin 2 virus was added to the first tube containing 180 μL of nOPV2c1-295, pipetted and 20 μL of the mixture transferred to the second tube containing 180 μL of nOPV2c1-295 mixed and 20 μL of the mixture transferred to next tube, the same procedure was repeated until the last tube, the last 20 μL of the mixture was discarded, and the RNA was extracted from each dilution and subjected to qosRT-PCR analysis as described above.

### 2.5. qmosRT-PCRs for Virus Genome Copy Number Calculation

To quantify the genome copy (GC) number in each spiked sample (Sabin 2 spiked in nOPV2c1-295) a quantitative multiplex one-step RT-PCR (qmosRT-PCR) was used. Briefly, the qmosRT-PCR reactions were prepared in 96-well optical plates in a final volume of 25 μL using 2 μL of RNA of test and control samples (2 μL of DNA-plasmid for reference standards) and QuantiFast Multiplex RT-PCR Kit (QIAGEN, Valencia, CA, USA). The RNAs of Sabin 2 and nOPV2c1-295 viruses were used as positive controls, and water was used as negative control. Plasmid containing genome of Sabin 2 virus with known GC number was used as standard reference for extrapolation of GC for Sabin 2 in test samples, and plasmid containing genome of nOPV2c1-295 virus with known GC number was used as standard reference for extrapolation of GC number for nOPV2c1-295 in the spiked samples.

Control samples were run in quadruplets, reference standard samples in duplicates, and the test samples were run in triplicate repeats. The specific primer pairs and probes used for each virus are: for Sabin 2, Sab2-538F (forward primer), Sab2-605R (reverse primer) and Sab2PrbFAM2 (TaqMan probe) (Table 1), and for nOPV2-c1, nOPV2-538F (forward primer), 5′TTGAGCAGGCAGCTGCAAC3′, nOPV2-605R (reverse primer), 5′GTAGTCGGTTTCGCCATT3′ and nOPV2PrbYAK2 (TaqMan probe), YAK-5′AGCAGCCAGCCTGT3′-ZEN/3IaBkFQ.

The primers were used at a concentration of 0.8 μM each and the TaqMan probes were used at concentration 25 nM each. The qmosRT-PCR procedure was performed using real-time PCR System ViiA7 (Applied Biosystems, Foster City, CA, USA) at the following thermal cycling conditions: one cycle incubation for 20 min at 50 °C and 5 min at 95 °C, followed by 45 cycles, each consisting of 15 s at 95 °C, 15 s at 55 °C and 30 s at 60 °C.

### 2.6. Illumina Sequencing

**Figure 1:**
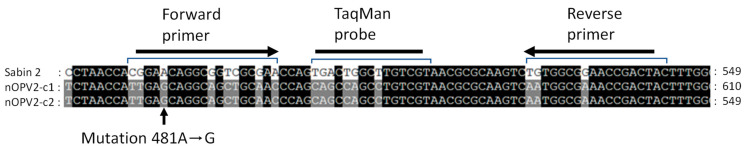
Nucleotide sequences of domain V region of nOPV2 and Sabin 2 viruses. Genomic location of primers and TaqMan probe used for detection and quantitation of Sabin 2 virus in nOPV2 vaccine and mutation 481A→G are indicated.

RNA was extracted from the nOPV2 lots provided by Bio Farma (see above) and nOPV2c1-295 as described above and used for RNA library preparation. The RNA library was prepared using the NEBNext Ultra II RNA Library Prep Kit for Illumina (New England BioLabs, Ipswich, MA, USA). Fragmentation (to generate about 500 nt RNA fragments) and priming were performed in one reaction using the buffer provided in the kit. The first and second strands of DNA were synthesized according to the manufacturer’s protocol. The resulting DNA fragments were ligated to Illumina paired end adaptors, then amplified using 12 cycles of PCR with multiplex indexed primers and purified by magnetic beads (Agencourt AMPure PCR purification system, Beckman Coulter, Brea, CA, USA). After analyzing the libraries for the size and quality with BioAnalyzer (Agilent Technologies, Santa Clara, CA, USA), sequencing was performed using MiSeq (Illumina, San Diego, CA, USA) producing 250 nt paired end reads. The raw sequencing reads were analyzed by an in-house developed specialized platform High-performance Integrated Virtual Environment (HIVE) [28]. The RNA sequence of nOPV2-c1 (GenBank accession number: MZ245455) virus was used as genome reference for HIVE alignment and mutations profiling of sequencing reads generated from nOPV2 lots. To differentiate Sabin 2 virus from nOPV2 we targeted specifically the introduced 18 SNPs in domain V of nOPV2 viruses (Figure 1).

## 3. Results

### 3.1. Design of Specific Primers and Probes for Sabin 2 Virus Detection and Quantification

To identify and quantify Sabin 2 virus in nOPV2 samples, a qosRT-PCR procedure was developed. Domain V of the Internal Ribosome Entry Site (IRES) element in the 5′-Utranslated Region (5′ UTR) was selected to design specific primers and a TaqMan probe. This region contains the attenuating mutation 481-A that can mutate to produce a virulent revertant 481-G (Figure 1) [3,4]. As part of the development of nOPV2, this region was modified to increase its genetic stability by eliminating the possibility of reversion to neurovirulence through single-nucleotide changes [18]. The differences between this modified region called S15 and Sabin 2 virus were used as a genetic marker for the design of primers and a TaqMan probe for detection and quantitation Sabin 2 virus in nOPV2 (Figure 1). The primers and the probe are described in Table 1 and Figure 1.

### 3.2. Linearity, Specificity and Limit of Detection of the Assay for Detection of Sabin 2 RNA

**Figure 2:**
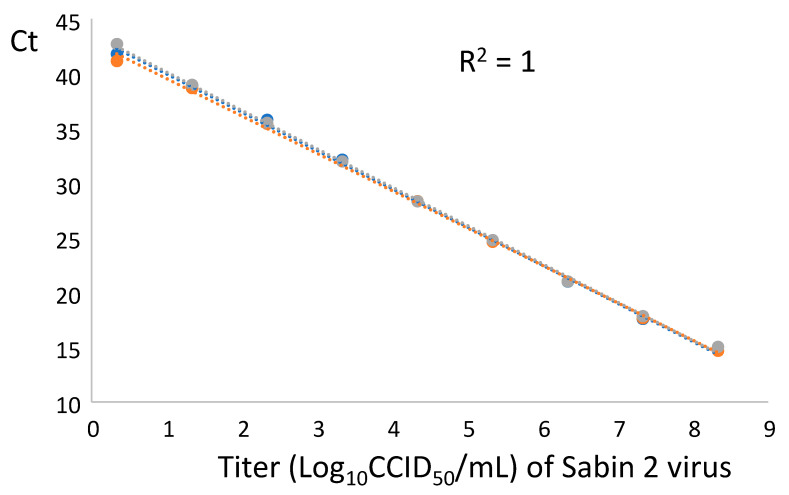
The three repeats of qosRT-PCR analysis (each repeat is presented in one line) of RNA diluted samples: RNA was extracted from Sabin 2 virus (with titer of 8.34 Log_10_ CCID_50_/mL), serially diluted 10-times using 10-fold steps and subjected to qosRT-PCR analysis. The R^2^ is around 1 for each repeat of the analyzed samples.

To evaluate the linearity and sensitivity of the RT-PCR in absence of nOPV2 background, a Sabin 2 strain with titer of 8.34 Log_10_ CCID_50_/mL was subjected to RNA extraction as described above and the RNA was serially diluted 10-times using 10-fold steps in a total volume of 100 μL. The original and diluted RNA samples were tested, and the resulting Ct values were averaged (mean) for each dilution and plotted against the respective Sabin 2 titers expressed as log_10_ CCID_50_/mL as shown in Figure 2. This result showed that the assay was able to detect RNA of 2 CCID_50_/mL (Relative to the titer of the original sample and RNA dilution) and had a very broad linearity range of at least 8 log_10_ (from 8.34 to 0.34 Log_10_ CCID_50_/mL). RNA from a sample with the lowest titer analyzed (0.034 Log_10_ CCID_50_/mL) was undetectable.

**Figure 3:**
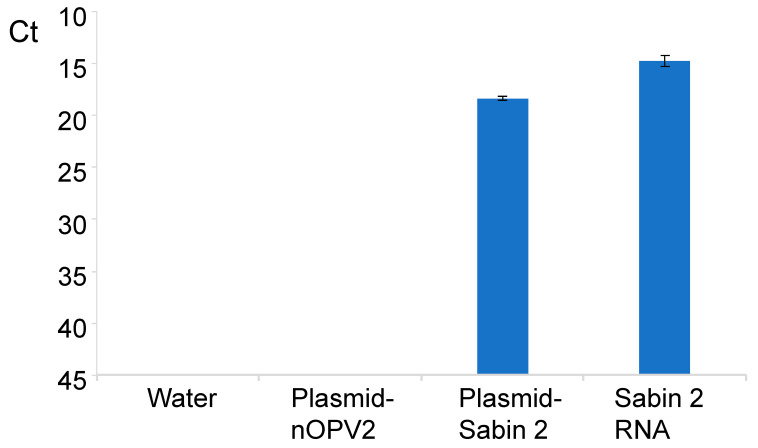
Specific Sabin 2 virus identification with qosRT-PCR demonstrated by using Sabin 2 RNA and plasmid containing Sabin 2 genome (positive controls), and plasmid containing the nOPV2-c1genome and water (negative controls).

In the same run, Sabin 2 RNA (8.34 Log_10_ CCID_50_/mL) and a plasmid containing Sabin 2 genome (0.1 ng/mL) were run as positive controls, while another plasmid containing the nOPV2-c1genome (0.1 ng/mL) and water were used as negative controls. The controls were run in quadruplet repeats, and the test and standard reference samples were tested in triplicates. The results of positive and negative controls are presented in Figure 3. The method could detect all positive controls and none of negative controls was detected; this result demonstrates that the method is very specific for the detection of Sabin 2 strain.

### 3.3. Repeatability of the RT-PCR for Detection Sabin 2 Strain

Three Sabin RNA samples with equivalent viral titers of 2.16, 21.63 and 216.27 CCID_50_/mL that were used in the above experiment were also used to study the repeatability of the assay. The samples were analyzed in triplicate repeats in three runs and on two different days by the same operator. Table 2 summarizes the averages for each triplicate in each run. The results demonstrate that the assay for detection of Sabin 2 strain generated consistent results for all three analyzed samples. Results of these experiments showed no significant differences in titers repeatedly obtained on the same run, different run, same day or on different days. The observed variations mostly did not exceed 2-fold difference with few exceptions, especially for sample 9, which had the lowest concentration that could be detected by the assay as shown above (~2 CCID_50_/mL). Briefly, the Sabin 2 detection assay yielded consistent and reproducible results.

### 3.4. Detection of Sabin 2 Virus in the Presence of nOPV2 Virus

**Figure 4:**
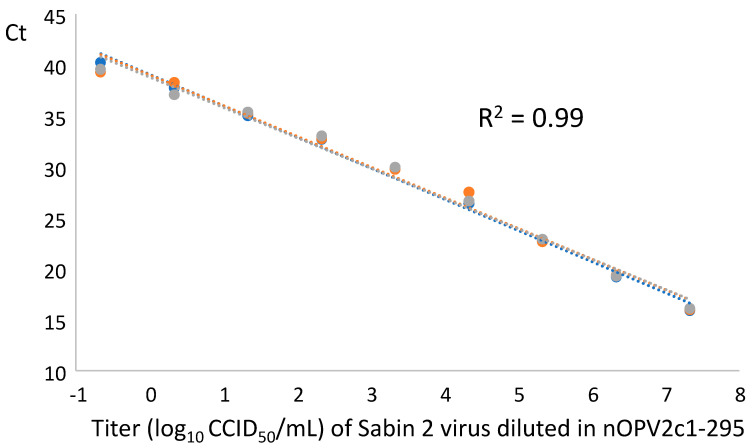
The three repeats of qosRT-PCR analysis (each repeat is presented in one line) of Sabin 2 virus in presence of prevalent amounts of nOPV2: Sabin 2 virus (titer 8.34 log_10_ CCID_50_/mL) was serially diluted in 10-fold steps in nOPV2c1-295 virus (titer of 8.07 log_10_ CCID_50_/mL). All diluted samples were subjected to RNA extraction and then to the assay analysis. The R^2^ is around 0.99 for each repeat of the analyzed samples.

To check the ability of the simple qosRT-PCR assay to detect Sabin 2 virus in the presence of large amounts of nOPV2 virus, Sabin 2 virus with the titer 8.34 log_10_ CCID_50_/mL was serially diluted in 10-fold steps in nOPV2c1-295 virus suspension with the titer of 8.07 log_10_ CCID_50_/mL (as described above in materials and methods paragraph). All diluted samples were subjected to RNA extraction and then to the assay for detection Sabin 2 contamination in nOPV2c1-295 as separate samples in triplicate repeats. The results showed that the presence of nOPV2 virus with high titer did not interfere with the assay that was able to detect Sabin 2 virus at levels of 0.22 CCID_50_/mL which correspond to traces of Sabin 2 virus in nOPV2 (2 × 10^−7^% of Sabin 2 in nOPV2 suspension) (Table 3 and Table 4). A linear fit of the data had R^2^ equal 0.99 and over a range of 8 log_10_ (Figure 4). All negative controls including nOPV2c1-295 virus and nOPV2-c1 plasmid were negative and the positive control was positive (Table 3), confirming the specificity of the assay.

To compare the percentages of the spiked Sabin 2 virus in nOPV2c1-295 prepared based on CCID_50_ to those based on genome copy (GC) numbers we analyzed the spiked samples with qmosRT-PCR as described above and the resulting GC numbers were used to calculate the percentages of the Sabin 2 in the samples (Table 4). The average GC:CCID_50_ ratios for the Sabin 2 virus used to prepare the spiked samples is about 990. In addition, we subjected these samples to Illumina next generation sequencing (NGS) and the percentages of Sabin 2 in samples were calculated as the average percentages of the 18 SNPs in domain V (Figure 1) as described above and shown in Table 4. These results showed qmosRT-PCR (GC multiplex quantification) could detect 2 × 10^−5^% and Illumina-sequencing detect 0.2% of Sabin 2 virus in the spiked samples, and both methods generated results that correlate well with the percentages of the spiked samples based on CCID_50_ with R^2^ equal of 0.98 and 1.00 respectively. On the other hand, the simple quantification of Sabin 2 with qosRT-PCR detected 0.22 CCID_50_/mL of Sabin 2 in the presence of nOPV2, which correspond to 198 GC/mL as the GC:CCID_50_ ratio for Sabin 2 virus is around 990 (Table 3). Therefore, the qosRT-PCR assay is more sensitive than NGS and specific for detection of Sabin 2 in the presence of relevant nOPV2 amounts.

### 3.5. Analysis of nOPV2 Vaccine Lots for the Presence of Sabin 2 Contamination

To validate the assay for detection of Sabin 2 contamination in nOPV2 stocks, different lots of nOPV2 candidate 1 and candidate 2 vaccines (manufactured by Bio Farma, Indonesia) that are mentioned above (See material and methods paragraph) and presented in Table 5 were tested on three different days, one run per day. Sabin 2 virus was not detected in the nOPV2 vaccines lots; however, in a few samples we observed one of the three repeats with Ct ≥ 40, which are still negative results, while positive controls were positive and negative controls were negative. These results were confirmed by NGS analysis that did not reveal the presence of SNPs in domain V (Figure 1) of nOPV2 lots in comparison to nOPV2-c1 sequence (nOPV2-c1 and nOPV2-c2 have the same domain V sequence) used as a reference for bioinformatic analysis, confirming the absence of Sabin 2 virus in these nOPV2 lots. This result and the data above show that the assay for detection of Sabin 2 contamination in nOPV2 stocks can be used for quality control of nOPV2 lots to detect and quantify Sabin 2 virus as a possible contaminant.

## 4. Discussion

Vaccines are the most efficient tools for preventing, controlling and even eradicating infectious diseases. However, contamination can occur at various stages of vaccine preparation, resulting from the unintentional introduction of extraneous agents present in raw materials or introduced during the manufacturer’s process.

As stated above, the nOPV2 was created by introducing targeted genetic changes to the Sabin 2 strain that further stabilize the attenuated phenotype. Since the original Sabin 2 virus can easily convert to circulating vaccine-derived poliovirus type 2 (cVDPV2), it is important to ensure that batches of nOPV2 do not contain Sabin 2 virus if handled in the same manufacturing facility. Therefore, a sensitive and specific assay for detection of Sabin 2 strain is useful for quality control of the nOPV2 vaccine.

The previous molecular methods were designed for specific detection of Sabin strains in clinical and environmental samples but cannot distinguish Sabin strains from their genetically modified nOPV derivatives [20,22,23,24,25]. Here we describe the assay for specific detection and quantitation of Sabin 2 virus even in the presence of relevant amounts of nOPV2 virus.

The following provisional layout and acceptance criteria are proposed: the assay for detection of Sabin 2 contamination in nOPV2 RNA samples are tested in triplicate along with reference–Sabin 2 RNA serially diluted in 10-fold steps (with at least 10^6^ to 10^1^ of the relative CCID_50_/mL), blank (water) and nOPV2 plasmid DNA and/or nOPV2 RNA as negative controls, and Sabin 2 RNA and/or Sabin 2 plasmid DNA as positive controls. All the samples are tested in triplicates or quadruplicates (for controls) in the same 96-well plates by qosRT-PCR that specifically detects Sabin 2 virus and differentiates it from the nOPV2. The qosRT-PCR run is considered valid if all the following conditions are met: R^2^ of the standard curve is more or equal 0.95, for three out of four positive control repeats Ct values are less or equal to 40, and for at least three out of four negative control repeats have Ct values undetermined (or Ct ≥ 40). The test sample is positive if at least two out of three repeats have Ct values less than 40. In this work the Ct 40 was chosen as threshold. However, to establish a real Ct threshold needs further work to ensure that the assay is specific and works under different conditions, and in different hands and labs.

The assay proved to be very sensitive, detecting about of 0.2 CCID_50_/mL an equivalent of 198 GC/mL (as the used Sabin 2 virus has a GC:CCID_50_ ratio of about 990) of Sabin 2 virus diluted in nOPV2 suspension, and 198 GC/mL corresponds to 0.396 GC/reaction which is in line with having reached the maximum theoretical limit of one GC detected per reaction. The limit of detection may vary from virus to virus depending upon the GC:CCID_50_ ratio for each virus. No cross-amplification was observed with nOPV2 and a plasmid that contains nOPV2 genome (Figure 3, Table 3 and Table 5). In addition, the assay for detection of Sabin 2 contamination in nOPV2 stocks had a wide linearity range of at least 8 log_10_ and R^2^ value of dose-response curve of about 1.0. The assay generated consistent results as shown by testing Sabin 2 RNA samples with low concentrations that corresponds to the virus titer of 2–200 CCID_50_/mL (Table 2). However, small variations were observed in sample 9 which has titer of about 2 CCID_50_/mL (Table 2), although the variations are mostly related to the small sample volume (2 µL) used per reaction and difficulty of ensuring absolute sample homogeneity.

The presence of high titer of nOPV2 in the tested samples had no effect on the specificity of the assay, in contrast the sensitivity which appeared to have been improved (Figure 4 and Table 3 and Table 4). Even considering a detection limit of 2 CCID_50_ in a bulk lot of over 7 log CCID_50_, the method will exclude levels of contamination of 0.1 CCID_50_ per 10^5^ CCID_50_ dose. Data supporting 50% infectious dosages for Sabin 2 are limited, but for risk assessment purposes have been estimated at 100–1000 CCID_50_; this suggests the method provides a substantial margin of safety.

This assay was evaluated using different nOPV2 lots (Bio Farma, Indonesia). No Sabin 2 virus was detected in these lots and the results were confirmed by NGS (Table 5). Based on the analysis of domain V region of the viral genome where there are 18 nucleotide differences between Sabin 2 and nOPV2 genomes (Figure 1), this assay can be used for quality control of nOPV2 lots to detect and quantify Sabin 2 virus as a possible contaminant. For that purpose, it will need further validation to account for location, analysts, reagents, and equipment used to perform the assay.

In conclusion, the assay for detection Sabin 2 contamination in nOPV2 stocks described in this communication offers a simple and rapid method for the detection and quantitation of Sabin 2 virus either individually or in the presence of nOPV2 virus. The assay for Sabin 2 contamination in nOPV2 stocks is designed specifically to be applied during manufacture of nOPV2 vaccine, for quality control to detect Sabin 2 virus as a potential contaminant in nOPV2 vaccine.

## Figures and Tables

**Table 1:** Primers and TaqMan probe for detection and quantitation of Sabin 2 virus in nOPV2.

Name	Seq 5′-->3′	Sabin 2 Location	Tm (Basic)	Size (nt)	Amplicon Size (bp)
Sab2-605R (Reverse)	GTAGTCGGTTCCGCCACA	544–527	57	18	67
Sab2-538F (Forward)	CGGAACAGGCGGTCGCGAA	477–495	58	19	
Sab2PrbFAM2 (TaqMan Probe)	FAM-TGACTGGCTTGTCGT-ZEN//3IaBkFQ/	500–514	42	15	

**Table 2:** Analysis of repeatability of results generated by the qosRT-PCR Sabin 2 contamination assay.

Day	Run	Sabin 2 Virus		
		Sample 7	Sample 8	Sample 9
1	1	215.94 220.22 216.14 240.09	19.47 12.99 19.86 14.87	2.68 3.90 2.03 1.16
2	2	190.05 249.62 300.66 297.63	12.43 15.83 17.27 8.84	3.59 4.42 4.16 5.01
	3	269.98 280.74 249.19 289.66	19.41 10.69 18.93 16.35	9.32 3.18 3.62
Average		251.66	15.58	3.92
STDEV		36.44	3.68	2.10

Note: The expected titers of samples are as follow: 216.27 CCID_50_/mL for sample 7, 21.63 CCID_50_/mL for sample 8 and 2.16 CCID_50_/mL for sample 9. SD: standard deviation error, Avg: Average.

**Table 3:** Detection and quantification of Sabin 2 virus serially diluted in nOPV2c1-295.

Sample Names	% of Spiked Sabin 2 Virus in nOPV2c1-295	Sabin 2 Titers (Log_10_ CCID_50_/mL)	Average Ct ± STDEV
1	16.98	7.34	16.00 ± 0.11
2	1.91	6.34	19.28 ± 0.08
3	1.94 × 10^−1^	5.34	22.79 ± 0.12
4	1.94 × 10^−2^	4.34	26.87 ± 0.58
5	1.94 × 10^−3^	3.34	29.88 ± 0.10
6	1.94 × 10^−4^	2.34	32.83 ± 0.20
7	1.94 × 10^−5^	1.34	35.23 ± 0.19
8	1.94 × 10^−6^	0.34	37.71 ± 0.61
9	1.94 × 10^−7^	−0.66	39.72 ± 0.48
10	1.94 × 10^−8^	−1.67	UD
11	1.94 × 10^−9^	−2.67	UD
12	1.94 × 10^−10^	−3.67	UD
Water	Negative Controls	NA	UD
nOPV2c1-295 virus		8.07	UD
nOPV2 plasmid		1 ng/µL	UD
Sabin 2 plasmid	Positive Control	0.1 ng/µL	16.93 ± 0.34

Note: UD; undetermined, NA; Not applicable.

**Table 4:** Correlation between the Sabin 2 percentages calculated from the spiked samples based on CCID50 and genome copy number, and by using Illumina-sequencing.

Expected % (CCID_50_)	GC# %	NGS %
	R^2^ = 0.98	R^2^ = 1.00
16.98	32.02	18.02
1.91	7.59	1.94
1.94 × 10^−1^	1.32	0.23
1.94 × 10^−2^	1.02 × 10^−1^	UD
1.94 × 10^−3^	4.21 × 10^−3^	UD
1.94 × 10^−4^	5.67 × 10^−5^	UD
1.94 × 10^−5^	1.77 × 10^−7^	UD
1.94 × 10^−6^	UD	UD
1.94 × 10^−7^	UD	UD
1.94 × 10^−8^	UD	UD
1.94 × 10^−9^	UD	UD
1.94 × 10^−10^	UD	UD

Note: GC#, genome copy number, NGS; Illumina sequencing.

**Table 5:** Analysis of nOPV2 lots by qosRT-PCR assay for detection Sabin 2 contamination.

nOPV 2 Lots	Day 1		Day 2		Day 3		NGS Result	
	Run1: Ct	Result	Run 2: Ct	Result	Run 3: Ct	Result	Sabin 2	nOPV2
**Monovalent nOPV2-c1**								
nPol2 016C-c1	UD	Negative	UD	Negative	UD	Negative	Negative	Positive
	UD		UD		UD			
	UD		UD		40			
nPol2 018C-c2	UD	Negative	UD	Negative	UD	Negative	Negative	Positive
	UD		UD		UD			
	UD		UD		UD			
**Drug product nOPV2-c1**								
2060119C	UD	Negative	UD	Negative	UD	Negative	Negative	Positive
	UD		UD		UD			
	UD		UD		UD			
2060219C	UD	Negative	UD	Negative	UD	Negative	Negative	Positive
	UD		UD		UD			
	UD		UD		UD			
2060319C	UD	Negative	UD	Negative	UD	Negative	Negative	Positive
	UD		UD		UD			
	UD		UD		UD			
**Monovalent nOPV2-c2**								
nPOL 2056C	43	Negative	UD	Negative	UD	Negative	Negative	Positive
	UD		UD		UD			
	UD		UD		UD			
nPOL 2038C	UD	Negative	40	Negative	UD	Negative	Negative	Positive
	UD		UD		UD			
	UD		UD		UD			
**Negative controls**								
Water	UD	Negative	UD	Negative	UD	Negative	NA	NA
	UD		UD		UD			
	UD		UD		UD			
	UD		UD		UD			
nOPV2-Plasmid	UD	Negative	UD	Negative	UD	Negative	NA	NA
	UD		UD		UD			
	UD		UD		UD			
	UD		UD		UD			
**Positive control**								
Sabin 2-plasmid	18	Positive	14	Positive	15	Positive	NA	NA
	17		14		15			
	18		13		14			
	18		14		15			

Note: nOPV2-plasmid; plasmid containing nPOV2-c1 genome, Sabin2-plasmid; plasmid containing Sabin 2 genome, NA; not applicable, NGS: Illumina sequencing, UD; undetermined.

## Data Availability

All relevant data are within the paper.

## References

[1] Laassri M., Lottenbach K., Belshe R., Wolff M., Rennels M., Plotkin S., Chumakov K. (2005). Effect of Different Vaccination Schedules on Excretion of Oral Poliovirus Vaccine Strains. J. Infect. Dis..

[2] Modlin J.F. (1991). Mucosal immunity following oral poliovirus vaccine and enhanced potency inactivated poliovirus vaccine immunization. Pediatr. Infect. Dis. J..

[3] Dunn G., Begg N.T., Cammack N., Minor P.D. (1990). Virus excretion and mutation by infants following primary vaccination with live oral poliovaccine from two sources. J. Med. Virol..

[4] Laassri M., Lottenbach K., Belshe R., Rennels M., Plotkin S., Chumakov K. (2006). Analysis of Reversions in the 5′-Untranslated Region of Attenuated Poliovirus after Sequential Administration of Inactivated and Oral Poliovirus Vaccines. J. Infect. Dis..

[5] WHO (2019). Two Out of Three Wild Poliovirus Strains Eradicated. Global Eradication of Wild Poliovirus Type 3 Declared on World Polio Day.

[6] Chard A.N., Datta D., Tallis G., Burns C.C., Wassilak S.G.F., Vertefeuille J.F., Zaffran M. (2020). Progress Toward Polio Eradication—Worldwide, January 2018–March 2020. MMWR Morb. Mortal. Wkly. Rep..

[7] Faden H., Modlin J.F., Thoms M.L., McBean A.M., Ferdon M.B., Ogra P.L. (1990). Comparative Evaluation of Immunization with Live Attenuated and Enhanced-Potency Inactivated Trivalent Poliovirus Vaccines in Childhood: Systemic and Local Immune Responses. J. Infect. Dis..

[8] This Week. Polio This Week as of 18 October 2017. This Week—GPEI—Global Polio Eradication Initiative. http://polioeradication.org/polio-today/polio-now/this-week/.

[9] Burns C.C., Diop O.M., Sutter R.W., Kew O.M. (2014). Vaccine-Derived Polioviruses. J. Infect. Dis..

[10] Kew O.M., Sutter R.W., De Gourville E.M., Dowdle W.R., Pallansch M.A. (2005). Vaccine-derived polioviruses and the endgame strategy for global polio eradication. Annu. Rev. Microbiol..

[11] Platt L.R., Estívariz C.F., Sutter R.W. (2014). Vaccine-Associated Paralytic Poliomyelitis: A Review of the Epidemiology and Estimation of the Global Burden. J. Infect. Dis..

[12] Baicus A. (2012). History of polio vaccination. World J. Virol..

[13] John J., Giri S., Karthikeyan A.S., Iturriza M., Muliyil J., Abraham A., Grassly N.C., Kang G. (2014). Effect of a single inactivated poliovirus vaccine dose on intestinal immunity against poliovirus in children previously given oral vaccine: An open-label, randomised controlled trial. Lancet.

[14] Rubin J., Ottosen A., Ghazieh A., Fournier-Caruana J., Ntow A.K., Gonzalez A.R. (2017). Managing the Planned Cessation of a Global Supply Market: Lessons Learned From the Global Cessation of the Trivalent Oral Poliovirus Vaccine Market. J. Infect. Dis..

[15] Brickley E.B., Strauch C.B., Wieland-Alter W.F., Connor R.I., Lin S., Weiner J.A., Ackerman M.E., Arita M., Oberste M.S., Weldon W.C. (2018). Intestinal Immune Responses to Type 2 Oral Polio Vaccine (OPV) Challenge in Infants Previously Immunized with Bivalent OPV and Either High-Dose or Standard Inactivated Polio Vaccine. J. Infect. Dis..

[16] Thompson K.M., Duintjer Tebbens R.J. (2017). Lessons from the Polio Endgame: Overcoming the Failure to Vaccinate and the Role of Subpopulations in Maintaining Transmission. J. Infect. Dis..

[17] Wright P.F., Connor R.I., Wieland-Alter W.F., Hoen A.G., Boesch A.W., Ackerman M.E., Oberste M.S., Gast C., Brickley E., Asturias E.J. (2016). Vaccine-induced mucosal immunity to poliovirus: Analysis of cohorts from an open-label, randomised controlled trial in Latin American infants. Lancet Infect. Dis..

[18] Konopka-Anstadt J.L., Campagnoli R., Vincent A., Shaw J., Wei L., Wynn N.T., Smithee S.E., Bujaki E., Yeh M.T., Laassri M. (2020). Development of a new oral poliovirus vaccine for the eradication end game using codon deoptimization. NPJ Vaccines.

[19] Macadam A.J., Ferguson G., Stone D.M., Meredith J., Knowlson S., Auda G., Almond J.W., Minor P.D. (2006). Rational design of genetically stable, live-attenuated poliovirus vaccines of all three serotypes: Relevance to poliomyelitis eradication. J. Virol..

[20] Van der Avoort H.G., Hull B.P., Hovi T., Pallansch M.A., Kew O.M., Crainic R., Wood D.J., Mulders M.N., Van Loon A.M. (1995). Comparative study of five methods for intratypic differentiation of polioviruses. J. Clin. Microbiol..

[21] Buonagurio D.A., Coleman J.W., Patibandla S.A., Prabhakar B.S., Tatem J.M. (1999). Direct detection of Sabin poliovirus vaccine strains in stool specimens of first-dose vaccinees by a sensitive reverse transcription-PCR method. J. Clin. Microbiol..

[22] De L., Nottay B., Yang C.F., Holloway B.P., Pallansch M., Kew O. (1995). Identification of vaccine-related polioviruses by hybridization with specific RNA probes. J. Clin. Microbiol..

[23] Kilpatrick D.R., Yang C.-F., Ching K., Vincent A., Iber J., Campagnoli R., Mandelbaum M., De L., Yang S.J., Nix A. (2009). Rapid group-, serotype-, and vaccine strain-specific identification of poliovirus isolates by real-time reverse transcription-PCR using degenerate primers and probes containing deoxyinosine residues. J. Clin. Microbiol..

[24] Laassri M., Dipiazza A., Bidzhieva B., Zagorodnyaya T., Chumakov K. (2013). Quantitative one-step RT-PCR assay for rapid and sensitive identification and titration of polioviruses in clinical specimens. J. Virol. Methods.

[25] Laassri M., Mee E.T., Connaughton S.M., Manukyan H., Gruber M., Hernandez C.R., Minor P.D., Schepelmann S., Chumakov K., Wood D.J. (2018). Detection of bovine viral diarrhoea virus nucleic acid, but not infectious virus, in bovine serum used for human vaccine manufacture. Biologicals.

[26] van der Werf S., Bradley J., Wimmer E., Studier F.W., Dunn J.J. (1986). Synthesis of infectious poliovirus RNA by purified T7 RNA polymerase. Proc. Natl. Acad. Sci. USA.

[27] Manukyan H., Rodionova E., Zagorodnyaya C., Lin T.-L., Chumakov K., Laassri M. (2019). Multiplex PCR-based titration (MPBT) assay for determination of infectious titers of the three Sabin strains of live poliovirus vaccine. Virol. J..

[28] Simonyan V., Mazumder R. (2014). High-Performance Integrated Virtual Environment (HIVE) Tools and Applications for Big Data Analysis. Genes.

